# Profile of severe maternal morbidity cases by nationality in Colombia: analysis of national surveillance data, 2021

**DOI:** 10.3389/fgwh.2026.1746675

**Published:** 2026-05-14

**Authors:** John Camilo Garcia Uribe, Aníbal Arteaga Noriega, Ana Maria Zuleta, Paula Andrea Serna

**Affiliations:** 1 Facultad de Enfermeria, Grupo de investigación GIPECS, Universidad de Antioquia, Medellín, Colombia; 2 Grupo de investigación Salud Familiar y Comunitaria, Corporacion Universitaria Remington Facultad de Ciencias de la Salud, Medellín, Colombia

**Keywords:** extreme maternal morbidity, maternal mortality, migrant health, migration, pregnancy, sociodemographic factors

## Abstract

Severe maternal morbidity (SMM) surveillance data allow characterization of critical obstetric events. Evidence on SMM case profiles among foreign vs. Colombian women in Colombia remains limited. This study aims to To compare sociodemographic and clinical characteristics among women notified as severe maternal morbidity (SMM) cases in Colombia in 2021. We conducted a secondary cross-sectional analysis of a 2021 national surveillance database restricted to women classified as severe maternal morbidity (SMM) cases. Nationality (Colombian vs. foreign) was the exposure; outcomes were the presence (yes/no) of specific complications within the SMM event. We estimated crude and adjusted prevalence ratios (PR/aPR) using Poisson regression with log link and robust variance, adjusting for maternal age, gravidity, socioeconomic stratum, and late entry to prenatal care; complete-case analysis was used (effective *N* = 2,676). A total of 3,210 patient records were included, of which 86.8% were Colombian and 13.2% were foreings. Sociodemographic and clinical variables were analyzed using descriptive and inferential statistics. The results show that the main causes of SMM were preeclampsia (66.3%), obstetric hemorrhage (23.5%), and cardiovascular failure (43.5%). A higher incidence of late entry into prenatal care was observed among foreing women. These findings may contribute to the implementation of health strategies aimed at mitigating the impact of SMM on vulnerable populations.

## Introduction

The World Health Organization (WHO) defines extreme maternal morbidity (EMM) as: “A woman who nearly dies, but survives complications that arose during pregnancy, childbirth, or the 42 days after the end of pregnancy” (1). Such complications must be life-threatening and require immediate attention to prevent death (2).

Maternal morbidity surveillance emerged in the 1990s with the aim of identifying the reasons for maternal deaths (MD), risk factors, and individual characteristics, health services, and the social and cultural environment where the event occurs, in order to design corrective and preventive measures. However, it was not until 2012 that SMM was incorporated into the National Health Institute's surveillance system, and over the years, attempts have been made to standardize the criteria used for the detection and reporting of SMM cases (2). The surveillance model in Colombia uses the case definition of the Latin American Federation of Obstetrics and Gynecology Societies (FLASOG), which includes any woman who meets one or more inclusion criteria related to signs and symptoms of a specific disease, with organ failure or dysfunction, and with the patient's established management (3).

To discuss extreme maternal morbidity, it is necessary to refer to the causes of maternal death worldwide, which, according to the WHO, are mainly due to conditions such as severe postpartum hemorrhage, severe preeclampsia/eclampsia, sepsis, and complications during childbirth (uterine rupture) or following an abortion (4). Between 2000 and 2023, the maternal mortality ratio (i.e., the number of maternal deaths per 100,000 live births) declined globally by approximately 40%. In 2023, just over 90% of all maternal deaths occurred in low- and lower-middle-income countries (4). It is estimated that both globally and in Latin America, there are around 12–14 cases of extreme maternal morbidity for every case of maternal death. In 2012, the incidence of extreme maternal morbidity was between 0.31% and 4.93% (5).

Regarding extreme maternal morbidity in Colombia in 2020, there was an increase in cases compared to previous years. However, the main causes continue to be hypertensive disorders associated with pregnancy in first place and obstetric hemorrhage in second place, as evidenced in previous years. This increase is due to the contingency caused by SARS-CoV-2 infection, not precisely because of maternal infection with the new virus, but because of limited access to health services during the contingency period (6). For 2020, there were 24,251 reported cases per 619,504 live births (7).

In 2019, Antioquia recorded 1,727 cases of extreme maternal morbidity, with a rate of 23.7 cases per 100,000 live births, lower than Colombia, with 36.5 cases per 1,000 live births in the same period. The subregion of the department with the highest incidence was Valle de Aburrá with 60.1%. Additionally, the most common event was severe preeclampsia, followed by postpartum hemorrhage. Of these, 50% of cases were in the 20–29 age group (20).

It is important to emphasize that around 90% of maternal deaths in Latin America and the Caribbean are preventable, which creates a need for information and monitoring of the phenomenon (6). For the year 2030, the sustainable development goals set by the Pan American Health Organization (PAHO) defined within goal 3 a target to reduce maternal mortality. For this reason, it is important to characterize the population and identify the factors associated with the development of these pathologies so that, in the future, we can define the factors on which to intervene to prevent their development (8).

An additional challenge facing the country and the region is the migration of people from Venezuela. In August 2023, it was estimated that there were 1.5 million Venezuelan women living in Colombia, of whom only 810,000 had some form of affiliation with the General Social Security System for Health (SGSSS) (9). Therefore, the inability to access health services leads to significant difficulties in controlling comorbidities, limited access to contraceptive methods (21), and fewer prenatal checkups.

Based on the above, this research aims to compare sociodemographic and clinical characteristics among women notified as severe maternal morbidity (SMM) cases in Colombia in 2021, stratified by nationality (Colombian vs. foreign).

## Materials and methods

We conducted a secondary analysis of a national surveillance database from 2021. The analytic sample was restricted to women classified as cases of severe maternal morbidity (SMM)/extreme maternal morbidity (EMM) according to the surveillance definition in effect for that year. Because the dataset includes only SMM cases, this study does not estimate risk factors for developing SMM in the general obstetric population; instead, it compares characteristics and complication profiles among women with SMM, stratified by nationality

The primary exposure was nationality as recorded in the surveillance notification. Colombian nationality was used as the reference category (“non-migrant”), and foreign nationality as the comparison category (“migrant”). We acknowledge that nationality is an imperfect proxy for migration status and does not capture migration trajectory, duration of residence, legal status, or displacement context. Nationality recorded in the surveillance notification (Colombian vs. foreign) was used as a proxy for migration-related status.

Given that all participants were SMM cases, study outcomes were defined as the presence (yes/no) of specific complications occurring within the SMM event. Each complication was operationalized as a binary variable based on the corresponding surveillance fields (see Supplementary Table X for variable definitions). These outcomes represent within-case complication profiles rather than incidence of SMM.

Severe maternal morbidity (SMM) was defined based on disease-specific criteria documented in the surveillance notification: severe preeclampsia, eclampsia, sepsis/septic shock, and severe obstetric hemorrhage (blood loss >500 mL accompanied by hemodynamic instability). Likewise, patients whose extreme maternal morbidity was not clearly defined or cases that were outside the established time range were excluded.

We summarized sociodemographic and obstetric characteristics available in the dataset, including maternal age (continuous), number of pregnancies/gravidity (categorized), socioeconomic stratum (categorized), and late entry to prenatal care (yes/no), which were included *a priori* as potential confounders in adjusted models.

For data analysis, categorical variables were described using frequencies and percentages. Maternal age was summarized as a continuous variable (mean and standard deviation or median and interquartile range, as appropriate). We compared foreign-national vs. Colombian SMM cases using chi-square tests or Fisher's exact tests for categorical variables, as appropriate. For each complication outcome, we estimated crude prevalence ratios (PR) to quantify differences in complication prevalence between nationality groups.

To obtain adjusted measures of association, we fitted generalized linear models with a Poisson distribution, log link, and robust (sandwich) variance estimators to estimate adjusted prevalence ratios (aPR) and 95% confidence intervals (95% CI) for each complication outcome. Models were adjusted for maternal age (continuous), number of pregnancies/gravidity (categorical), socioeconomic stratum (categorical), and late entry to prenatal care (yes/no). Analyses were conducted in jamovi using the GAMLj module (Generalized Linear Model), specifying a Poisson family, log link, and robust variance estimation; exponentiated coefficients (Exp[B]) were reported as PR/aPR.

No imputation was performed. Multivariable models were estimated using complete-case analysis. The effective sample size for adjusted models was *N* = 2,676 (as implemented in the software), reflecting exclusion of observations with missing values in any modeled variable

## Ethical considerations

In accordance with national regulations and institutional policies for secondary analysis of anonymized public health surveillance records, informed consent was not required. No procedures were performed involving human participants beyond routine public health notification.

## Results

Clinical and sociodemographic characteristics of patients with extreme maternal morbidity, Antioquia 2021.

Descriptive analysis of the sociodemographic and clinical variables of the population. 86.8% of the women were Colombian nationals, while 13% were migrants. In terms of socioeconomic status, 62.4% belonged to strata 1 and 2, and 37.5% to strata 3 or higher. 23.2% of women had late access to prenatal care (PNC).

In terms of parity, 45% were primigravidas. Regarding obstetric complications, preeclampsia was the most frequent, affecting 66.3% of pregnant women. Eclampsia occurred in 4.1%, septic shock in 6.7%, obstetric hemorrhage in 23.5%, and uterine rupture in 1.1% (Table 1).

**Table 1:** Clinical characteristics of the study population.

Variable	*N*	Frequency
Nationality		
Colombia	2,786	86.80%
Foreign	417	13%
Socioeconomic stratum		
Stratum 1 and 2	1,671	62.40%
Stratum 3 and above	1,005	37.50%
No data	534	
Late entry to ANC*		
Yes	746	23.20%
No	2,464	76.80%
Parity		
1	1,444	45%
2	878	27.40%
3	445	13.90%
4	219	6.80%
5 or more	224	6.97%
Eclampsia		
Yes	133	4.10%
Preeclampsia		
Yes	2,127	66.30%
Septic shock		
Yes	216	6.70%
Obstetric hemorrhage		
Yes	754	23.50%
Uterine rupture		
Yes	36	1.10%

* Antenatal Care.

### Clinical characteristics of extreme maternal morbidity by nationality

Foreign-national women had a higher prevalence of severe preeclampsia (74.3% vs. 65.0%; crude PR 1.15, 95% CI 1.08–1.22; *p* < 0.001) and eclampsia (6.8% vs. 3.7%; crude PR 1.92, 95% CI 1.25–2.94; *p* = 0.003). In contrast, severe obstetric hemorrhage was less prevalent among foreign-national cases (17.7% vs. 24.4%; crude PR 0.67, 95% CI 0.51–0.87; *p* = 0.002). No statistically significant difference was observed for septic shock (6.4% vs. 6.8%; crude PR 0.93, 95% CI 0.62–1.40; *p* = 0.750).

Regarding complications and selected clinical/history variables, coagulation failure was less prevalent among foreign-national cases (12.0% vs. 17.9%; crude PR 0.63, 95% CI 0.46–0.85; *p* = 0.003), whereas cardiac failure showed a non-significant trend toward higher prevalence (47.6% vs. 42.9%; crude PR 1.22, 95% CI 0.99–1.50; *p* = 0.066). No statistically significant differences were found for uterine rupture, renal failure, hepatic failure, central nervous system failure, or respiratory failure.

Markers of prenatal care access differed notably by nationality. Foreign-national cases more frequently had late entry to prenatal care (37.3% vs. 21.1%; crude PR 1.79, 95% CI 1.53–2.04; *p* < 0.001) and reported no prenatal care visits (17.7% vs. 12.7%; crude PR 1.48, 95% CI 1.12–1.96; *p* = 0.005). Additionally, a history of previous cesarean section was less prevalent among foreign-national cases (56.1% vs. 63.6%; crude PR 0.88, 95% CI 0.82–0.95; *p* = 0.003), while the proportion in socioeconomic strata 1–3 was high in both groups (99.6% vs. 97.8%; crude PR 1.02, 95% CI 0.95–1.09; *p* = 0.042) (Table 2).

**Table 2:** Clinical/social characteristics and maternal morbidity.

Variable	Colombian *n* (%)	Foreign national *n* (%)	Crude PR (95% CI)*	*p*-value
SMM subtype				
Severe preeclampsia	1,812 (65.0)	315 (74.3)	1.15 (1.08–1.22)	<0.001
Eclampsia	104 (3.7)	29 (6.8)	1.92 (1.25–2.94)	0.003
Severe obstetric hemorrhage	679 (24.4)	75 (17.7)	0.67 (0.51–0.87)	0.002
Septic shock	189 (6.8)	27 (6.4)	0.93 (0.62–1.40)	0.750
Complications and clinical/history variables				
Uterine rupture	31 (1.1)	5 (1.2)	1.01 (0.41–2.78)	0.904
Cardiac failure	1,195 (42.9)	202 (47.6)	1.22 (0.99–1.50)	0.066
Renal failure	206 (7.4)	23 (5.4)	0.72 (0.46–1.12)	0.142
Hepatic failure	325 (11.7)	45 (10.6)	0.90 (0.64–1.25)	0.527
Central nervous system failure	89 (3.2)	12 (2.8)	0.89 (0.48–1.64)	0.689
Respiratory failure	171 (6.1)	20 (4.7)	0.76 (0.47–1.23)	0.249
Coagulation failure	498 (17.9)	51 (12.0)	0.63 (0.46–0.85)	0.003
Personal background				
Primigravida	1,268 (45.5)	176 (41.5)	0.91 (0.77–1.07)	0.123
Previous cesarean section	1,771 (63.6)	238 (56.1)	0.88 (0.82–0.95)	0.003
Late entry to prenatal care	588 (21.1)	158 (37.3)	1.79 (1.53–2.04)	<0.001
No prenatal care visits	353 (12.7)	75 (17.7)	1.48 (1.12–1.96)	0.005
Socioeconomic stratum 1–3	2,352 (97.8)	270 (99.6)	1.02 (0.95–1.09)	0.042

Crude prevalence ratios (PR) and 95% confidence intervals (CI) were estimated comparing foreign-national vs. Colombian SMM cases (reference = Colombian). *p*-values correspond to chi-square tests.

* Confidence Interval.

In the multivariable Poisson regression model with robust variance (Table 3), foreign nationality was independently associated with a higher prevalence of severe preeclampsia among SMM cases (aPR 1.39, 95% CI 1.12–1.72; *p* = 0.003), after adjustment for maternal age, gravidity, socioeconomic stratum, and late entry to prenatal care. Maternal age was inversely associated with severe preeclampsia (aPR 0.992 per year, 95% CI 0.987–0.998; *p* = 0.005). Compared with socioeconomic stratum 1, stratum 2 (aPR 1.11, 95% CI 1.02–1.21; *p* = 0.015) and stratum 3 (aPR 1.34, 95% CI 1.05–1.71; *p* = 0.017) were associated with higher prevalence of severe preeclampsia. Gravidity categories showed non-significant trends relative to primigravida/first pregnancy (gesta 2: aPR 1.08, *p* = 0.069; gesta 3: aPR 1.10, *p* = 0.077), and late entry to prenatal care was not statistically associated with severe preeclampsia in the adjusted model (aPR 1.07, 95% CI 0.98–1.16; *p* = 0.124).

**Table 3:** Multivariable poisson regression (robust variance) for severe preeclampsia among SMM cases.

Variable	aPR	95% CI	*p*-value
Nationality (Foreign vs. Colombian)	1.39	1.12–1.72	0.003
Maternal age (years, continuous)	0.992	0.987–0.998	0.005
Gravidity: 2 vs. 1	1.08	0.99–1.17	0.069
Gravidity: 3 vs. 1	1.10	0.99–1.23	0.077
Gravidity: 4 vs. 1	1.11	0.96–1.29	0.143
Gravidity: ≥5 vs. 1	1.08	0.93–1.26	0.315
Socioeconomic stratum: 2 vs. 1	1.11	1.02–1.21	0.015
Socioeconomic stratum: 3 vs. 1	1.34	1.05–1.71	0.017
Socioeconomic stratum: ≥4 vs. 1	1.11	0.41–2.97	0.834
Late entry to prenatal care (Yes vs. No)	1.07	0.98–1.16	0.124

aPR, adjusted prevalence ratio; CI, confidence interval. Estimates obtained from Poisson regression with log link and robust (sandwich) variance. Reference categories: Colombian nationality, gravidity = 1, socioeconomic stratum = 1, and no late entry to prenatal care. Models estimated using complete-case analysis.

In the adjusted Poisson regression model with robust variance (Table 4), foreign nationality was associated with a higher prevalence of eclampsia among SMM cases compared with Colombian nationality (aPR 1.98, 95% CI 1.66–2.35; *p* < 0.001), after adjustment for maternal age, gravidity, socioeconomic stratum, and late entry to prenatal care. None of the covariates included in the model showed statistically significant associations with eclampsia (all *p* > 0.05), including maternal age (aPR 1.00 per year, *p* = 0.631) and late entry to prenatal care (aPR 1.00, *p* = 0.975).

**Table 4:** Multivariable poisson regression (robust variance) for eclampsia among SMM cases.

Variable	aPR	95% CI	*p*-value
Nationality (Foreign vs. Colombian)	1.98	1.66–2.35	<0.001
Maternal age (years, continuous)	1.00	0.997–1.01	0.631
Gravidity: 2 vs. 1	1.00	0.94–1.07	0.968
Gravidity: 3 vs. 1	1.01	0.92–1.10	0.856
Gravidity: 4 vs. 1	0.99	0.88–1.12	0.910
Gravidity: ≥5 vs. 1	1.01	0.89–1.15	0.863
Socioeconomic stratum: 2 vs. 1	1.01	0.94–1.08	0.801
Socioeconomic stratum: 3 vs. 1	1.02	0.95–1.09	0.606
Socioeconomic stratum: 4 vs. 1	1.02	0.82–1.27	0.841
Socioeconomic stratum: ≥5 vs. 1	1.03	0.58–1.82	0.920
Late entry to prenatal care (Yes vs. No)	1.00	0.94–1.07	0.975

aPR, adjusted prevalence ratio; CI, confidence interval. Estimates obtained from Poisson regression with log link and robust (sandwich) variance. Models estimated using complete-case analysis.

Similarly, in the severe obstetric hemorrhage model (Table 5), foreign nationality was associated with a 74% increase in prevalence (aPR = 1.74; 95% CI: 1.46–2.07; *p* < 0.001), while stratum 3 showed a lower prevalence compared to stratum 1 (aPR = 0.90; 95% CI: 0.83–0.97; *p* = 0.004); age, number of pregnancies, and late admission were not significant. Taken together, these findings indicate that, among women with extreme maternal morbidity, migrant status is independently associated with a higher prevalence of severe hypertensive complications and obstetric hemorrhage, even after adjusting for relevant sociodemographic and obstetric factors.

**Table 5:** Multivariable poisson regression (robust variance) for severe obstetric hemorrhage among SMM cases.

Variable	aPR	95% CI	*p*-value
Nationality (Foreign vs. Colombian)	1.74	1.46–2.07	<0.001
Maternal age (years, continuous)	1.00	0.999–1.01	0.178
Gravidity: 2 vs. 1	0.98	0.91–1.05	0.529
Gravidity: 3 vs. 1	0.95	0.86–1.04	0.254
Gravidity: 4 vs. 1	0.96	0.84–1.09	0.491
Gravidity: ≥5 vs. 1	0.96	0.84–1.10	0.575
Socioeconomic stratum: 2 vs. 1	0.99	0.92–1.06	0.691
Socioeconomic stratum: 3 vs. 1	0.90	0.83–0.97	0.004
Socioeconomic stratum: ≥4 vs. 1	0.81	0.63–1.03	0.088
Socioeconomic stratum: ≥5 vs. 1	1.06	0.47–2.37	0.887
Late entry to prenatal care (Yes vs. No)	0.99	0.92–1.06	0.669

## Discussion

This study examined differences in clinical profiles and care-related characteristics among women notified as severe maternal morbidity (SMM) cases in 2021, stratified by nationality (foreign-national vs. Colombian). A key finding was the higher prevalence of delayed entry to prenatal care among foreign-national cases (37.3%) compared with Colombian cases (21.1%). Because the analysis is restricted to SMM cases, this result should be interpreted as a difference in prenatal care access within the SMM case population, rather than as an estimate of SMM risk. Prior work in Colombia has described barriers affecting timely access and continuity of maternal healthcare among migrant populations, including limitations related to health system affiliation/insurance, administrative and informational barriers, discrimination and stigmatization, and geographic and economic constraints, which may contribute to delayed initiation and fragmentation of prenatal and postpartum care (10, 11, 25).

We also observed differences in SMM subtypes by nationality. In crude comparisons, foreign-national cases showed a higher prevalence of severe preeclampsia and eclampsia than Colombian cases. In adjusted models, foreign nationality remained associated with higher prevalence of both severe preeclampsia (aPR 1.39, 95% CI 1.12–1.72) and eclampsia (aPR 1.98, 95% CI 1.66–2.35) among SMM cases, after accounting for maternal age, gravidity, socioeconomic stratum, and delayed entry to prenatal care. These findings align with reports of disproportionate maternal health burdens in migrant populations, potentially shaped by structural determinants such as poverty, social exclusion, and differential access to timely obstetric care (12, 13, 17). Mechanistic explanations (e.g., chronic stress pathways) (18) have been proposed in the literature, but such constructs were not measured in our dataset and should be considered hypothesis-generating in this context (14, 22).

Findings for severe obstetric hemorrhage and coagulation failure require careful interpretation. In crude analyses, severe obstetric hemorrhage and coagulation failure were less prevalent among foreign-national cases than among Colombian cases; however, the adjusted model indicated a higher prevalence of severe obstetric hemorrhage among foreign-national cases (aPR 1.74, 95% CI 1.46–2.07). This change in direction suggests confounding by measured covariates and/or differences introduced by complete-case analysis, and it underscores the need to report crude and adjusted results side-by-side and to describe missingness patterns explicitly. Differences in prior obstetric history such as previous cesarean delivery (19, 24) may partially contribute to hemorrhage-related complications through conditions like placenta previa or placenta accreta spectrum, although such diagnoses were not directly available in our dataset and cannot be confirmed here (15). In our data, previous cesarean section was less prevalent among foreign-national SMM cases than among Colombian cases.

Although the data correspond to 2021, they capture a defined post-peak period of regional migration and remain relevant because surveillance definitions and care pathways for obstetric emergencies have not fundamentally changed, national-level estimates of SMM case profiles in foreign nationals are scarce, and policy service planning frequently rely on the most recent complete surveillance year available. We nonetheless acknowledge that migration patterns and service access may have evolved since 2021 (23). Besides, national epidemiological surveillance systems in the country face operational challenges (e.g., underreporting, delays, and variable completeness), and these challenges may be more pronounced for migrant populations (16) due to barriers in access and documentation.

This study has limitations inherent to secondary analyses of routine surveillance data. First, underreporting and differential completeness of notification may occur, and this could affect comparisons by nationality. Second, nationality is a crude proxy for migration status; the dataset lacks key migration-related variables (e.g., regular/irregular status, duration of residence, and migration trajectory) as well as other potential confounders (e.g., detailed access measures, comorbidity profiles, and clinical severity markers). Third, we used complete-case analysis for multivariable models; if missingness was not completely at random particularly for socioeconomic variables estimates may be biased. Finally, some complications may be underdiagnosed where access to specialized testing is limited, potentially introducing differential misclassification. Despite these limitations, the findings highlight heterogeneity in clinical profiles and care-related characteristics among SMM cases by nationality and support the need for improved access and continuity of maternal care for vulnerable populations. Future research should incorporate richer migration and care-access measures and evaluate trends using more recent surveillance years to reflect evolving migration dynamics and health system changes.

## Conclusion

In this secondary analysis restricted to women notified as severe maternal morbidity (SMM) cases in 2021, foreign nationality was associated with differences in clinical profiles and care-related characteristics compared with Colombian nationality. Foreign-national SMM cases had higher adjusted prevalence of severe preeclampsia and eclampsia and were more likely to report delayed initiation of prenatal care. These results describe heterogeneity within SMM cases and should not be interpreted as estimates of risk factors for developing SMM in the general obstetric population. Strengthening timely access and continuity of antenatal and emergency obstetric care for vulnerable groups may help reduce preventable complications.

## Data Availability

The data analyzed in this study is subject to the following licenses/restrictions: the dataset belongs to the hospital. Requests to access these datasets should be directed to johnc.garcia@udea.edu.co.

## References

[1] Organización Panamericana de la Salud. Plan de Acción Para Acelerar la Reducción de la Mortalidad Materna y la Morbilidad Materna Grave: Estrategia de Monitoreo y Evaluación. Washington: Pan American Health Organization (PAHO) (2012). Available online at: https://iris.paho.org/handle/10665.2/49332 (Accessed November 18, 2025).

[2] Ministerio de Protección Social. Monitoring of Extreme Maternal Morbidity (EMM). Bogotá, Colombia: Ministerio de la Protección Social (2010). Available online at: https://www.minsalud.gov.co/sites/rid/Lists/BibliotecaDigital/RIDE/VS/PP/SM-Evaluacion-Modelo-vigilancia-morbilidad-materna-extrema.pdf (Accessed November 18, 2025).

[3] Ortiz EI Herrera E De La Torre A. Extreme maternal morbidity: a tracer event to improve the quality of obstetric care in Latin America. Colombia Medica (Cali, Colombia). (2019) 50(4):286–92. 10.25100/cm.v50i4.4197 32476694 PMC7232947

[4] Organización Mundial de la Salud. La Mortalidad Materna. World Health Organization. (2025). Available online at: https://www.who.int/es/news-room/fact-sheets/detail/maternal-mortality (Accessed November 18, 2025).

[5] Madeiro AP Rufino AC Lacerda ÉZ Brasil LG. Incidence and determinants of severe maternal morbidity: a transversal study in a referral hospital in teresina, piaui, Brazil. BMC Pregnancy Childbirth. (2015) 15:210. 10.1186/s12884-015-0648-3 26347370 PMC4562200

[6] Gutiérrez NR. Report on Extreme Maternal Morbidity. Colombia: Instituto Nacional de Salud de Colombia (2019). Available online at: https://www.ins.gov.co/buscador-eventos/Informesdeevento/MORBILIDAD%20MATERNA%20EXTREMA_2020.pdf (Accessed December 2, 2025).

[7] Ministerio de Salud y Protección Social. Health situation analysis (ASIS). Direct Epidemiol Demogr. (2020). Available online at: https://www.minsalud.gov.co/sites/rid/Lists/BibliotecaDigital/RIDE/VS/ED/PSP/asis-2020-colombia.pdf (Accessed December 5, 2025).

[8] Organización Panamericana de la Salud. Objetivos de Desarrollo Sostenible–OPS/OMS: Material Científico Técnico. (s. f.). Washington: Pan American Health Organization (PAHO). Available online at: https://www.paho.org/es/temas/determinantes-ambientales-salud/objetivos-desarrollo-sostenible-opsoms-material-cientifico (Accessed December 9, 2025).

[9] Ministerio de Salud y Protección Social. Observatorio Nacional de Migración y Salud. (s. f.). Bogotá: Ministerio de Salud y Protección Social. Available online at: https://www.sispro.gov.co/observatorios/onmigracionysalud/Paginas/Observatorio-Nacional-de-Migracion-y-Salud.aspx (Accessed December 12, 2025).

[10] García Uribe JC López MJ Betancur Penagos JC Figueroa LJ. Morbilidad materna e inequidad en tiempos de COVID-19: perspectivas bioéticas. Revista Colombiana De Bioética. (2024) 19(2):1–20. 10.18270/rcb.4823

[11] Olsson Wevel A. (Un)Universal Health Care for All: A Qualitative Study Exploring the Access to Health Care for Venezuelan Migrants in Medellín, Colombia. Lund: Lund University (2022). Available online at: http://lup.lub.lu.se/student-papers/record/9098992 (Accessed December 16, 2025).

[12] Bonilla-Tinoco LJ Aguirre-Lemus M Fernández-Niño JA. Venezuelan migrant population in Colombia: health indicators in the context of the sustainable development goals. F1000Res. (2020) 9:684. 10.12688/f1000research.24997.1

[13] Hung P Liu J Norregaard C Shih Y Liang C Zhang J Analysis of residential segregation and racial and ethnic disparities in severe maternal morbidity before and during the COVID-19 pandemic. JAMA Netw Open. (2022) 5(10):1–14. 10.1001/jamanetworkopen.2022.37711 PMC9585430 36264572

[14] Peredy T Greenwald M Fachon KD Danaher F Jasrasaria R Truong S Maternal mortality rates among im/migrant populations in the United States. Open J Obstet Gynecol. (2024) 14(08):1161–75. 10.4236/ojog.2024.148094

[15] Ghosh A Rani Ghosh S Das M. Risk factors and complications in pregnancies associated with placenta previa among admitted cases in FMCH. Int J Reprod Contracept Obstet Gynecol. (2023) 12(4):806–11. 10.18203/2320-1770.ijrcog20230774

[16] Angeleri S. A primary health care-anchored migrant right to health: insights from a qualitative study in Colombia. Health Hum Rights. (2024) 26(2):105–20. 39742212 PMC11683576

[17] Carvalho K Kheyfets A Lawrence B Moky A Harris L Abouhala S Examining the role of psychosocial influences on black maternal health during the COVID-19 pandemic. Matern Child Health J. (2022) 26(4):764–9. 10.1007/s10995-021-03181-9 34417954 PMC8379571

[18] Congreso de Colombia. Law 2244 of 2022: Recognizing the Rights of Women During Pregnancy, Labor, Childbirth, and Postpartum, and Establishing Other Provisions (“Law on Dignified, Respectful, and Humanized Childbirth”). Bogotá: Congreso de la República de Colombia (2022). Available online at: https://www.funcionpublica.gov.co/eva/gestornormativo/norma.php?i=189347 (Accessed December 22, 2025).

[19] Fenn MG Al Falahi M Al Hannai T Al Shukaili L Al Riyami N. Postpartum hemorrhage following vaginal and cesarean section deliveries at a single tertiary hospital: a five-year cross-sectional study. Oman Med J. (2024) 39(6):e695. 10.5001/omj.2024.116 40212577 PMC11983379

[20] Gobernación de Antioquia. Extreme Maternal Morbidity–MME. Medellín: Gobernación de Antioquia (2019–2020). Available online at: https://www.dssa.gov.co/images/documentos/MORBILIDAD_MATERNA.pdf (Accessed December 22, 2025).

[21] León Triana C. Salud sexual y reproductiva de la población migrante venezolana: más allá de la necesidad y la vulnerabilidad. Hojas de El Bosque. (2022) 8(15):17–22. 10.18270/heb.v8i15.4242

[22] Lueth AJ Allshouse AA Blue NM Grobman WA Levine LD Simhan HN Allostatic load and adverse pregnancy outcomes. Obstet Gynecol. (2022) 140(6):974–82. 10.1097/AOG.0000000000004971 36357956 PMC9712159

[23] Ministerio de Salud y Protección Social. Boletín Epidemiológico Semanal: Semana Epidemiológica 08 (20 al 26 de Febrero de 2022). Bogotá: Instituto Nacional de Salud (INS) (2022). Available online at: https://www.ins.gov.co/buscador-eventos/BoletinEpidemiologico/2022_Boletin_epidemiologico_semana_8.pdf

[24] Osayande I Ogunyemi O Gwacham-Anisiobi U Olaniran A Yaya S Banke-Thomas A. Prevalence, indications, and complications of caesarean section in health facilities across Nigeria: a systematic review and meta-analysis. Reprod Health. (2023) 20(1):81. 10.1186/s12978-023-01598-9 37268951 PMC10237076

[25] Rodríguez R Manuel J Jaramillo L María A. La afiliación al sistema de salud de personas migrantes venezolanas en Colombia. Población y Salud en Mesoamérica. (2021) 18(2):181–214. 10.15517/psm.v18i2.42795

